# Detection and quantification of a mycorrhization helper bacterium and a mycorrhizal fungus in plant-soil microcosms at different levels of complexity

**DOI:** 10.1186/1471-2180-13-205

**Published:** 2013-09-11

**Authors:** Florence Kurth, Katharina Zeitler, Lasse Feldhahn, Thomas R Neu, Tilmann Weber, Václav Krištůfek, Tesfaye Wubet, Sylvie Herrmann, François Buscot, Mika T Tarkka

**Affiliations:** 1 Department Soil Ecology, UFZ - Helmholtz Centre for Environmental Research, Theodor-Lieser-Str. 4, 06120 Halle/Saale, Germany; 2 Department River Ecology, UFZ - Helmholtz Centre for Environmental Research, Brückstraße 3a, 39114 Magdeburg, Germany; 3 Interfaculty Institute of Microbiology and Infection Medicine, University of Tübingen, Auf der Morgenstelle 28, Tübingen, Germany; 4 Biology Centre AS CR, v. v. i. - Institute of Soil Biology, Na Sádkách 7, 370 05 České Budějovice, Czech Republic; 5 German Centre for Integrative Biodiversity Research, University of Leipzig, Deutscher Platz 5, 04103 Leipzig, Germany; 6 Department of Community Ecology, UFZ - Helmholtz Centre for Environmental Research, Theodor-Lieser-Str. 4, 06120 Halle/Saale, Germany; 7 Institute of Biology, University of Leipzig, Johannisallee 21-23, 04103 Leipzig, Germany

**Keywords:** Streptomycetes, Oak, Real-time PCR, Rhizosphere, Microbial community

## Abstract

**Background:**

Host plant roots, mycorrhizal mycelium and microbes are important and potentially interacting factors shaping the performance of mycorrhization helper bacteria (MHB). We investigated the impact of a soil microbial community on the interaction between the extraradical mycelium of the ectomycorrhizal fungus *Piloderma croceum* and the MHB *Streptomyces* sp. AcH 505 in both the presence and the absence of pedunculate oak microcuttings.

**Results:**

Specific primers were designed to target the internal transcribed spacer of the rDNA and an intergenic region between two protein encoding genes of *P. croceum* and the intergenic region between the *gyrA* and *gyrB* genes of AcH 505. These primers were used to perform real-time PCR with DNA extracted from soil samples. With a sensitivity of 10 genome copies and a linear range of 6 orders of magnitude, these real-time PCR assays enabled the quantification of purified DNA from *P. croceum* and AcH 505, respectively. In soil microcosms, the fungal PCR signal was not affected by AcH 505 in the absence of the host plant. However, the fungal signal became weaker in the presence of the plant. This decrease was only observed in microbial filtrate amended microcosms. In contrast, the PCR signal of AcH 505 increased in the presence of *P. croceum*. The increase was not significant in sterile microcosms that contained plant roots.

**Conclusions:**

Real-time quantitative PCR assays provide a method for directly detecting and quantifying MHB and mycorrhizal fungi in plant microcosms. Our study indicates that the presence of microorganisms and plant roots can both affect the nature of MHB-fungus interactions, and that mycorrhizal fungi may enhance MHB growth.

## Background

Forests soils are highly complex ecosystems and soil microbes are known to have significant effects on plant diversity and productivity [1]. Most trees form a range of mutualistic associations with various filamentous fungi, these root-fungus associations are known as mycorrhizas. Mycorrhizal symbiosis improves plant nutrient acquisition and confers increased resistance to pathogens, while the fungus gains carbohydrates from its host plant [2]. The formation of mycorrhizas affects several aspects of plant physiology and also changes the nutritional and physical properties of the soil. The mycorrhizas and the external mycelia of symbiotic fungi (which together define the mycorrhizosphere) are colonised by bacteria, which may actively influence the growth of external fungal mycelia and mycorrhizal root colonisation. For instance, a group of bacteria known as Mycorrhization Helper Bacteria; MHB [3] stimulate the formation of mycorrhizas. At the time of writing, numerous bacterial strains from a wide range of major clades have been shown to have MHB-type functions in both arbuscular and ectomycorrhizal symbioses [4].

Bacteria can facilitate mycorrhization in various ways. In many cases, the positive effects stem from their ability to induce rapid expansion of the fungal mycelium e.g. [5]. Other important mechanisms include the alleviation of soil-mediated stress e.g. [6,7] and the formation of more extensive plant-fungus contacts by stimulating lateral root formation [8]. However, MHB do not always have positive effects on mycorrhiza formation and can exhibit fungus specificity in promoting symbioses [3]. While the effects of MHB on mycorrhizal fungi have been investigated extensively *in vitro*, the effects of the fungi on the MHB have largely been neglected. In their seminal work, Frey-Klett et al. [9] reported that the life span of the *Pseudomonas fluorescens* strain BBc6R8 was significantly prolonged by exposure to the EM-fungus *L. bicolor* S238N. This effect was attributed to the fungus because the survival of the bacterial strain was not affected by the presence of non-mycorrhizal roots.

Actinomycetes are frequent colonisers of mycorrhizospheres, rhizospheres and plant roots [10,11]. They are known for their antagonism against other microbial species [12,13] and are especially rich sources of antifungal compounds [14]. Depending on the circumstances, they can either inhibit or promote the formation of mycorrhizas reviewed in [11], and several actinomycete species exhibit MHB activity, Rhodococcus sp. [15], Streptomyces sp., [16-18]. Among the actinomycete MHB, the strain *Streptomyces* sp. AcH 505 has drawn most attention, since it forms unique interactions with fungi and plants. The extension of the fungal mycelium is promoted by the AcH 505 metabolite auxofuran [5], but the fungal biomass is simultaneously reduced due to the thinning of mycelium [19]. Schrey et al. [20] observed that co-cultivation of MHB *Streptomyces* sp. AcH 505 with *Amanita muscaria* and *Suillus bovinus* increased their rates of mycorrhization. However, co-cultivation with the same strain reduced the *in vitro* growth of *Hebeloma cylindrosporum*. This fungus-specificity is due to the differential sensitivity of the ectomycorrhizal fungi to the naphthoquinone antibiotic WS-5995 B, which is produced by AcH 505 [5] in addition to auxofuran. In the host plant, AcH 505 stimulated fine root formation [20] and facilitated root colonisation by suppressing the plant’s defensive responses [21]. However, while exposure to AcH 505 suppressed defensive responses at the root level, it increased the resistance to the causative agent of grey mould *Botrytis cinerea* at the leaf level. While previous studies on AcH 505 provided valuable information on its interactions with the host plant and ectomycorrhizal fungi, they were all based on *in vitro* experiments; to date, no studies on its effects in soil have been conducted.

The discovery of bacteria that promote the establishment and maintenance of mycorrhizas triggered a search for their mechanisms of actions, and a number of publications have described *in vitro* experiments on MHB-fungus interactions, e.g. [5,20,22]. However, much remains to be learned about how MHB-fungus interactions work under natural conditions and how they are affected by the host plant [4]. We therefore investigated the growth responses of AcH 505 and the mycorrhizal fungus *Piloderma croceum* using a soil-based culture system that was established for studying multitrophic interactions in oaks as part of the TrophinOak collaborative project [23], see also http://www.trophinoak.de. The pedunculate oak *Quercus robur* belongs to the *Fagaceae* family and is obligately ectomycorrhizal under natural conditions. It is host to several symbiotic fungi, including both basidio- and ascomycete species [24]. One of its notable symbiont is *Piloderma croceum*, which has become a model fungus for studying the formation of oak mycorrhizas [25]. In a preliminary investigation, we observed that AcH 505 promotes the formation of mycorrhizas in oak microcosms. The number of mycorrhizas per microcosm was counted prior to harvesting and was found to be slightly increased by inoculation with AcH 505 according to the test of equal proportions (p = 0.05).

The study conducted herein was conducted to assess i) whether the effects of Streptomyces sp. AcH 505 and the ectomycorrhizal fungus *Piloderma croceum* on one-another depend on the presence of a host plant, ii) the possible influence of the microbial community on both micro-organisms and iii) how the two micro-organisms influence each other.

For this purpose, AcH 505 and *P. croceum* were cultivated alone and together under four different culture conditions: in the presence of both the host plant (*Q. robur*) and soil microbes (represented by a microbial filtrate), in the presence of the host but not soil microbes, in the presence of soil microbes but no host plant, and in the presence of neither soil microbes nor the host. In microcosms including the plant rhizosphere as well as bulk soil samples were taken for quantification analysis. The experimental setup is summarised in Additional file 1.

The abundances of AcH 505 and *P. croceum* mycelia were estimated by quantitative real-time PCR [26]. Primers were designed to target an intergenic region of the AcH 505 genome, between the *gyrA* and *gyrB* genes. The abundance of eukaryotes in environmental samples can be determined using qPCR experiments targeting the highly variable internal transcribed spacer (ITS) regions of rDNA operons [27,28]. However, fungal genomes contain multiple copies of the ITS-region and the ITS copy number varies between fungal strains [29]. For *P. croceum* Raidl et al. [30] estimated about 150 ITS copies per dikaryotic cell. Thus, it can be beneficial to target single copy genes or intergenic regions rather than the ITS when quantifying fungi [29]. To compare the performance of these two approaches in fungal quantification, we designed novel ITS primers, as well as a primer pair that targets an intergenic region between two open reading frames (ORFs) in the *P. croceum* genome.

## Results

### Primer selection for real-time PCR and DNA extraction

**Figure 1:**
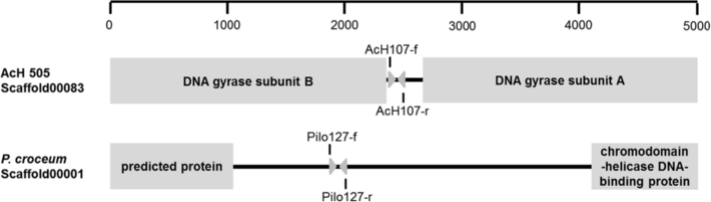
The target regions for the AcH107 and Pilo127 primer pairs.

**Figure 2:**
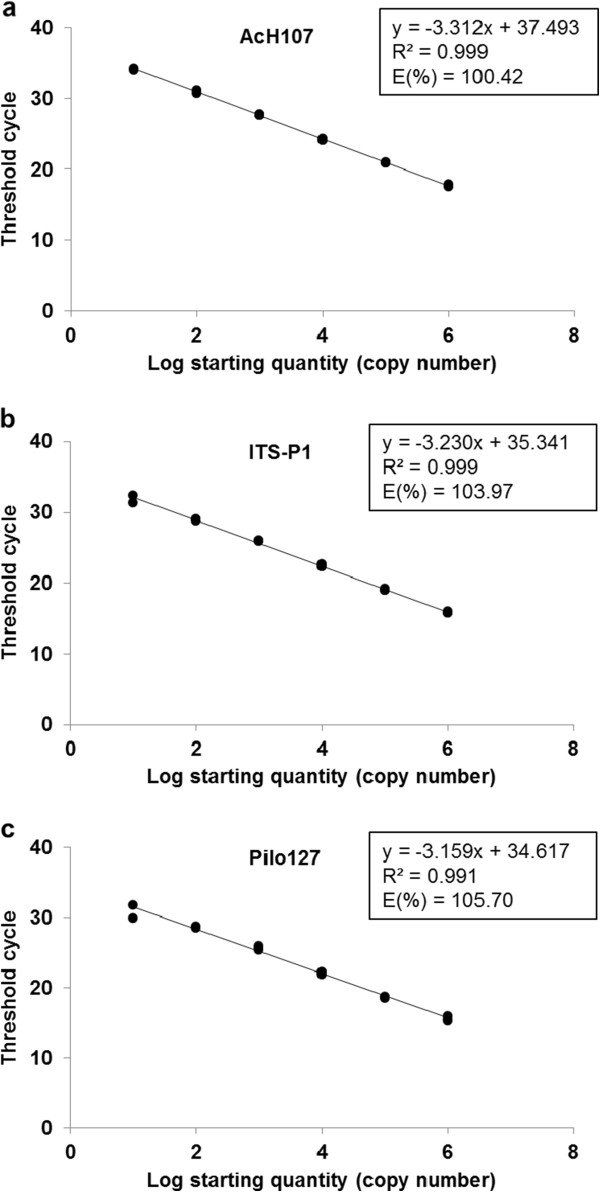
**Standard curves for the intergenic *gyrA/gyrB *region (a) and the ITS- (b) and intergenic region (c) in AcH 505 and *P. croceum *respectively.** Serial dilutions of plasmids with the target DNA insert were used in individual qRT-PCR assays to generate the standard curves. The R^2^ values, slopes and efficiencies are shown for each reaction.

Multiple templates were used to design specific primers for *Streptomyces* sp. AcH 505 including rRNA intergenic spacers, gene coding sequences, and regions between adjacent gene coding sequences. The specificity of each primer pair was evaluated by using them in real-time PCR experiments and analysing the melting curve of the resulting amplification products. The primer pair targeting the region between *gyrA* and *gyrB* genes exhibited specificity for AcH 505 sequences (i.e. it did not amplify sequences from *Piloderma croceum*, the soil microbe filtrate, or pedunculate oak DNA) as demonstrated by analysis of the melting curve for the PCR product it yielded. This primer pair had an efficiency of 76% as determined using a standard curve based on a serial two-fold dilution (see Additional file 2). The real-time PCR primers developed by Schubert et al. [31] for use with *P. croceum* samples were also tested but showed lower efficiency (Additional file 3). In addition, a novel ITS-specific primer pair was constructed based on the internal transcribed spacer region of *P. croceum* and primers were constructed to target the intergenic region between two ORFs based on the available genomic data for this species. Both primer pairs exhibited good efficiency and specificity for their respective amplification products (Additional files 4 and 5). The target regions for primer pairs AcH107 and Pilo127 are shown in Figure 1. Standard initial plasmid copy number versus cycle threshold (Ct) curves was used to estimate the frequencies of the target sequences in the DNA samples (Figure 2). The PCR fragments obtained using each primer pair were then cloned into plasmids. Serial plasmid dilutions were applied in each run to define the sensitivity of the method. As few as 10 copies per reaction were detected for each target sequence, and the initial copy numbers were linearly related to signal intensity over a range of 10^6^ to 10 copies of standard plasmid DNA. The limits of detection for real-time PCR with the AcH107-, ITSP1- and Pilo127 primers were determined by creating dilution series (in which the concentrations ranged from no dilution to dilution by a factor of 10^-5^) of bacterial and fungal DNA. All three primers yielded successful amplification at all dilutions above 10^-5^, corresponding to bacterial and fungal biomasses of approximately 15 and 2.5 ng, respectively (Additional file 6).

AcH 505 and *P. croceum* DNA from the microcosm soil were successfully amplified in all processed samples. The standard curves for the DNA preparations obtained for the different experimental treatments were all very similar, indicating that the samples did not differ in their contents of PCR-inhibiting substances.

### Quantification of *Streptomyces* sp. AcH 505 and *Piloderma croceum*

**Figure 3:**
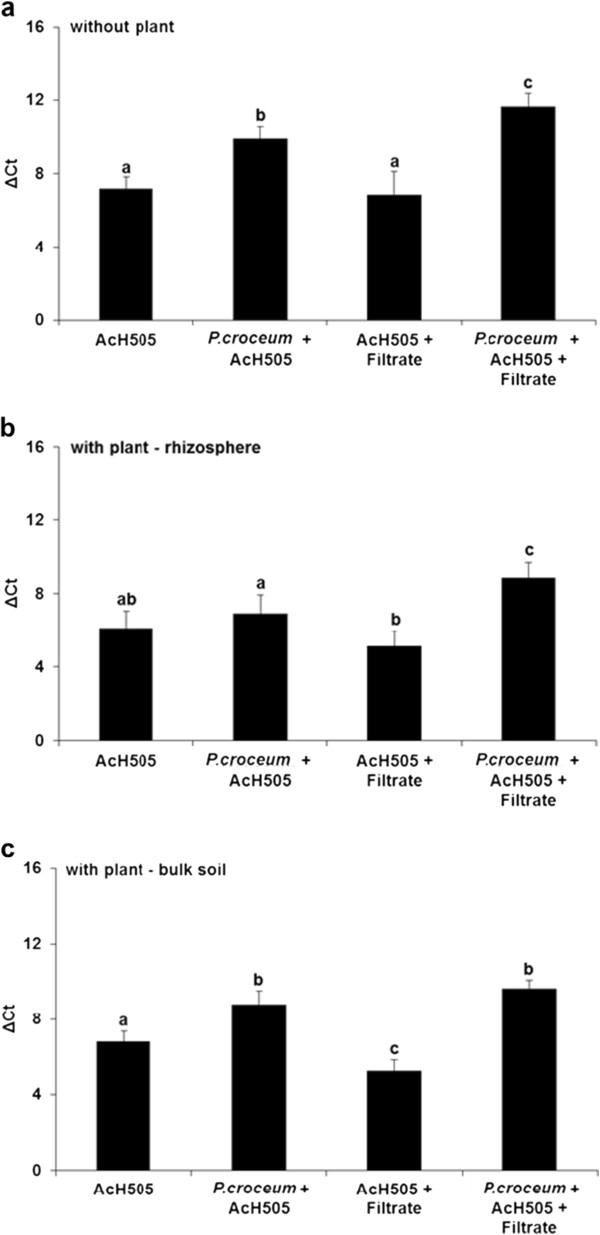
**Quantification of the mycorrhization helper bacterium *Streptomyces *sp. AcH 505 in soil microcosms.** The relative amounts of AcH 505 were measured by real-time quantitative PCR (qPCR) in the presence or absence of the mycorrhizal fungus *Piloderma croceum*, the soil microbial filtrate, and pedunculate oak microcuttings. In the presence of microcuttings quantification was performed with bulk soil as well as rhizosphere samples. The bars indicate the qPCR abundance of AcH 505 in the absence **(a)** and presence (rhizosphere **(b)** and bulk soil **(c)**) of the host plant. qPCR abundances are reported in terms of delta Ct values, which indicate the number of cycles at which the fluorescent signal exceeds the background level and surpasses the threshold established in the exponential section of the amplification plot. Error bars denote standard errors; bars with different letters are significantly different according to one-way ANOVA and the Tukey HSD test (*P* < 0.05). Note that co-inoculation with *P. croceum* stimulates the growth of AcH 505.

*P. croceum* significantly promoted the growth of AcH 505 in a culture system without oak microcuttings and in bulk soil samples in a culture system with oak (Figure 3a and c; see Additional file 7 for p-values). In the rhizosphere, *P. croceum* had no impact on AcH 505 in the sterile system, and the negative effects of the filtrate on AcH 505 that were only observed when the oak was present – in the rhizosphere as well as in the bulk soil -, could be released by the fungus (Figure 3b and c).

**Figure 4:**
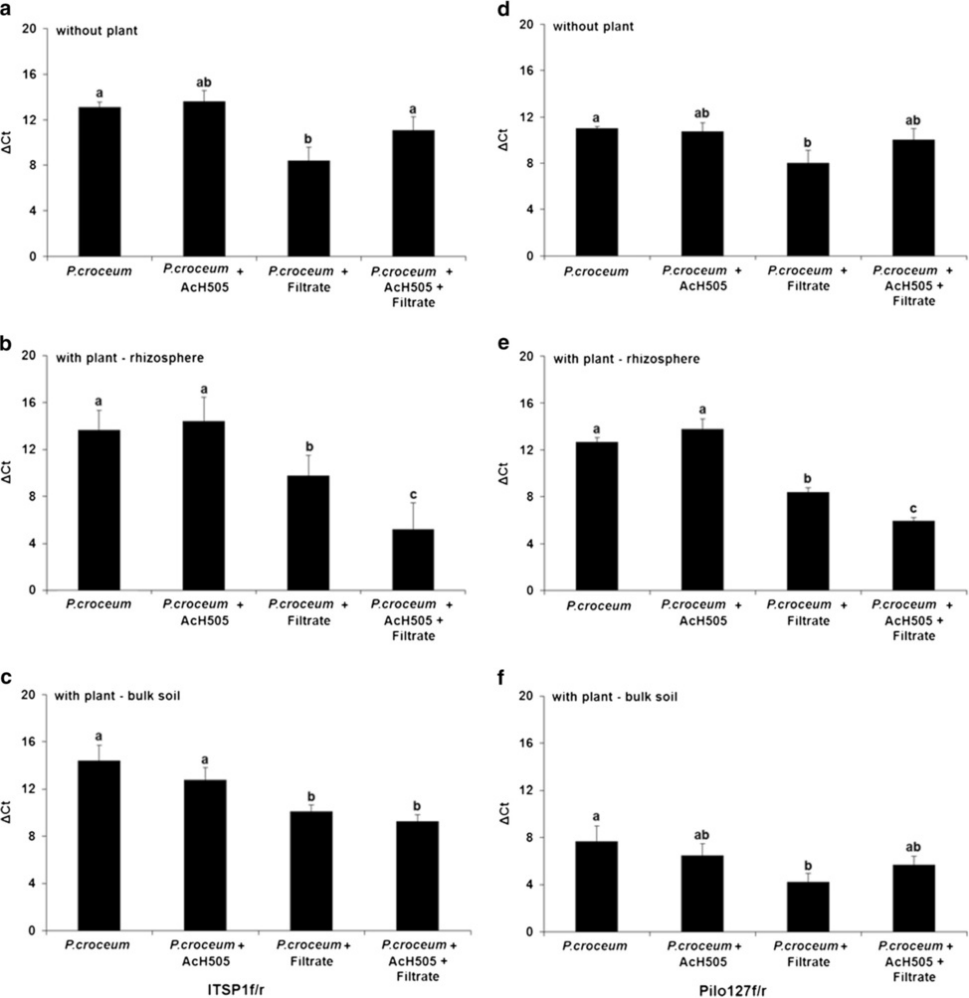
**Quantification of the ectomycorrhizal fungus *Piloderma croceum *in soil microcosms.** The relative amount of *P. croceum* mycelium was measured by real-time quantitative PCR (qPCR) in the presence or absence of *Streptomyces sp.* AcH 505, the soil microbial filtrate, and pedunculate oak microcuttings. In the presence of microcuttings quantification was performed with bulk soil as well as rhizosphere samples. The bars indicate qPCR abundances of *P. croceum* in the absence **(a,d)** and presence (rhizosphere **(b,e)** and bulk soil **(c,f)** of the host plant. Quantification was performed with the ITSP1f/r **(a,b,c)** and Pilo127f/r **(d,e,f)** primer pairs. The qPCR abundances are reported in terms of delta Ct values, which indicate the number of cycles at which the fluorescent signal exceeds the background level and surpasses the threshold established in the exponential region of the amplification plot. Error bars denote standard errors; bars with different letters are significantly different according to one-way ANOVA and the Tukey HSD test (*P* < 0.05). Note that the presence of the host plant modulates the responses of the microorganisms to one-another.

Treatment with the soil microbe filtrate following the initial application of the mycorrhizal fungus had a significant negative impact on the extraradical mycelium biomass of *P. croceum* in the culture system without pedunculate oak and in bulk soil in the presence of oak (Figure 4a,c,d and f). Co-inoculation with AcH 505 partially relieved this filtrate-based inhibition. In the presence of pedunculate oak, the filtrate’s inhibition of *P. croceum* was less pronounced (Figure 4b and e). However, AcH 505 inhibited *P. croceum* in the rhizosphere when the filtrate was applied to the microcosms. In conclusion, the presence of both soil microbes and oak microcuttings had significant effects on the interactions between AcH 505 and *P. croceum* in soil. Highly similar results were obtained using primer pairs that targeted the ITS region (ITSP1f/r) and the intergenic region (Pilo127f/r).

### Microscopic analysis of AcH 505 and *Piloderma croceum*

**Figure 5:**
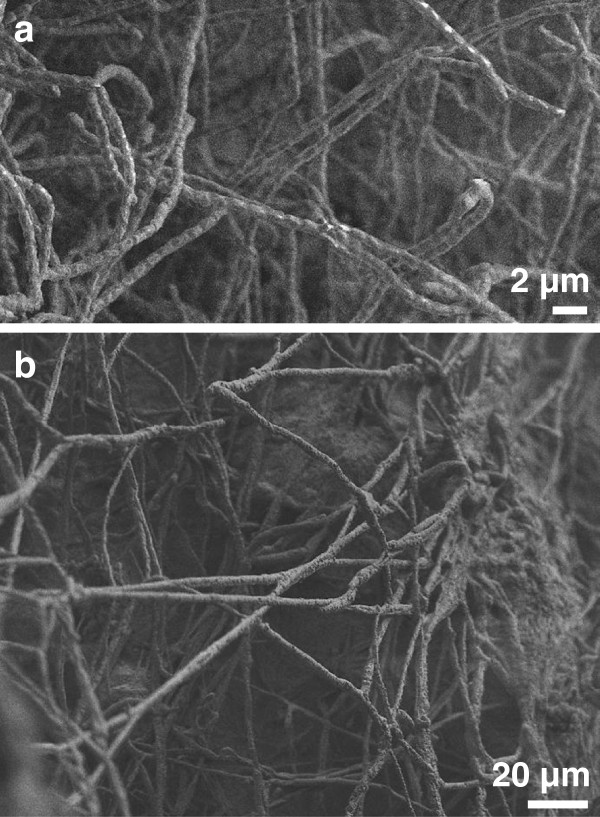
**Visualisation of *Streptomyces *sp. AcH 505 (a) and the *Piloderma croceum *(b) mycelium by scanning electron microscopy.**

AcH 505 and *P. croceum* were visualised within the soil microcosms using cryo-field emission scanning electron microscopy (Figure 5a and b; see Additional file 8 for a description of the method used). The bacterial filaments (Figure 5a) were easily distinguished by their small diameters (< 1 μm), branching and curvature, and segmentation by occasional septa. Fungal hyphae (Figure 5b) by contrast had an average diameter of 3 μm and were characterised by extensive branching. To visualise the interactions between the micro-organisms, *Streptomyces* sp. AcH 505 was labelled with green fluorescence protein, co-cultured with *P. croceum* on agar, and visualised by confocal laser scanning microscopy (see Additional files 9 and 10 for more details of these methods). The diameter of the AcH 505 filaments in the co-cultures was comparable to that observed by scanning electron microscopy in soil microcosms, and individual AcH 505 filaments often combined to form star-like bundles (Additional file 11). In addition, the AcH 505 filaments aligned on the surfaces of *P. croceum* hyphae. We did not detect adherence of AcH 505 on *P. croceum* in microcosms. The microscopic analyses demonstrate that both organisms can be visualised in soil microcosms.

## Discussion

Various hypotheses concerning the mechanisms that underpin the associations between mycorrhization helper bacteria (MHB), fungi and plants have been put forward based on the results of *in vitro* bioassays and cultures [20,22]. We have previously shown [5,19,21] that *Streptomyces* sp. AcH 505 is a fungus-specific MHB that produces fungus growth regulators and affects plant health and development. When tree roots were inoculated with a suspension of AcH 505 mycelia, significant stimulation of mycorrhiza formation was observed [19]. In the oak system, we also could find a slight increase in the number of mycorrhizas when the microcosm soil was inoculated with AcH 505. This was the first time when the mycorrhization helper effect was observed for AcH 505 in a soil based culture system. The present study further demonstrates the potential of this strain by casting light on its performance in a soil-vermiculate formulation, and shows that AcH 505 benefits from the presence of the mycorrhizal fungus.

### Specific detection of *Streptomyces* sp. AcH 505

Our initial experiments with AcH 505 were conducted using primers designed against the 16S-23S ribosomal DNA intergenic spacers and single copy genes. However, only the primers targeting the intergenic regions between protein-coding genes yielded specific amplification; the other tested primers were not suitable due to non-specific background amplification when used with samples that included soil microbe DNA. The ribosomal operon is present in multiple copies in streptomycetes [32], and different species within this genus can have different rDNA copy numbers. Moreover, the rate of rDNA sequence variation between the genomes of different *Streptomyces* strains is unknown. According to our preliminary analysis of the AcH 505 genome, the intergenic region between the *gyrA* and *gyrB* genes exists in a single copy and is thus an excellent target for specific quantification. The number of available genome data for different *Streptomyces* strains is increasing [33] and will enable the application of this simple and specific qPCR method for streptomycete quantification for even more bacterial isolates in the future.

### Comparable detection and quantification of *Piloderma croceum* by qPCR using two primer pairs

In basidiomycete fungi, the ribosomal genes are also present in multiple copies, and changes in the numbers of rRNA genes occur throughout the fungal life cycle [34]. Regions of rDNA are distributed as large tandem arrays, and intra-genomic variation in the length and the base distribution of rDNA sequences has been described [35]. Most qPCR quantification approaches in fungi are based on the internal transcribed spacer regions (ITS1 and ITS2) of the rDNA, since these are easily accessible by PCR and with their high copy number they allow a sensitive detection [27,28,31]. Due to the methodological constraints listed above, it can be argued that the use of single copy genes or intergenic regions between protein coding genes could allow for more accurate quantification of basidiomycete fungi. Our observations with *P. croceum* indicate, that at least this basidiomycete fungus is as effectively and quantitatively detected with primers targeting the ITS as with those constructed against an intergenic region between protein coding genes. Following the approach of Schubert et al. [31] we detected comparable ratios of ITS signal/mycelial biomass at different levels of fungal mycelium. In contrast, with another approach Raidl et al. [30] quantified the ITS copy number of *P. croceum* by using Taqman PCRs and by measuring the extent of mycelium from thin layers of sterile mycelium. To conclude, we could here clearly demonstrate how specific qPCR assays can be a powerful tool for elucidating the relative fungal and bacterial biomass in microcosm samples of varying complexity.

### Promotion of AcH 505 growth by *P. croceum* and response to soil microbial community

*P. croceum* promotes AcH 505 growth, which may indicate that the MHB feeds on fungal exudates. These include proteins, amino acids, and organic acids [36]; *P. croceum* is known to exude compounds such as oxalic and malic acid [37]. In ectomycorrhizal fungi such as *P. croceum*, trehalose is the primary storage sugar [38,39], and this disaccharide may be partially responsible for the selection of specific bacterial communities in mycorrhizospheres [4]. The positive impact of *P. croceum* on AcH 505 was more significant in microcosms amended with a microbe filtrate. This shows that competition by microbial community may influence the outcome of microbial interactions. Schlatter et al. [40] also reported, that the microbial community has an impact: *Streptomyces scabiei* DL87 promoted *Streptomyces lavendulae* DL93 in autoclaved, but not in field soil. In general, streptomycetes are competitive because they can derive nutrients from recalcitrant substrates, possess diverse resistance genes and are prolific producers of antagonistic secondary metabolites that inhibit the growth of their competitors [33,41]. It can also be concluded, that AcH 505 is a competitive streptomycete, as the strain was not affected by the microbe filtrate in the rhizospheres of plants.

### Fungal responses to soil microbial community and to AcH 505

The soil microbe filtrate inhibited *P. croceum*, and this inhibition could be due to competition for resources or space, or to antagonism [42]. The first of these possibilities, i.e. competitive inhibition, is perhaps more likely: Schrey et al. [43] obtained evidence that *P. croceum* may be particularly tolerant of antagonistic metabolites of Streptomycete isolates from Norway spruce – in an experiment conducted to determine the *in vitro* activity of *Piloderma* sp. mycorrhizas against seven fungi, *P. croceum* was the least severely affected fungus*.* In this study, *Streptomyces* affected the growth of *Piloderma* only under the influence of the microbial filtrate. This indicates that communities of soil microbes carry out a multitude of small-scale processes that can impact bacterium-fungus interactions [1,36].

### Plant rhizosphere reverses the outcome of AcH 505 - *P. croceum* interaction

Our observations of filtrate-amended microcosms demonstrated that the host plant has a strong effect on the MHB-fungus interaction: With the ITS primers it was observed that AcH 505 promoted *P. croceum* growth in the host plant’s absence, showed no significant impact in bulk soil, but inhibited the fungus in the rhizosphere. The numbers of ectomycorrhizal fine roots/seedling were not estimated. Thus, we cannot exclude local reductions in the numbers of ectomycorrhizal roots due to the AcH 505 treatment in the presence of soil microbe filtrate. Plants influence the composition and quantity of soil microbes by secreting products into the rhizosphere [44]. Root exudates contain compounds that can exert both stimulatory and inhibitory influences on the rhizosphere microbial community, changing its structure and composition [45]. Conversely, microbial products can induce plant root exudation [46]. AcH 505 influences its environment by the production of growth regulators [5]. In this work, the presence of oak rhizosphere might have led to increased production of antibiotics by AcH 505 which could perhaps cause the inhibition of *P. croceum* in the rhizosphere*.*

## Conclusions

Fungi and bacteria have established specific strategies for interacting with one-another with significant ecological consequences, as reviewed in [42]. Since one of the priorities in this context is to demonstrate the impact of particular organisms on each other, the development of methods for quantifying the abundance of bacteria and fungi in the presence of one-another and other potentially interfering microbes is essential. Our data suggest that significant interactions occur between AcH 505 and *P. croceum*. The competitive abilities of both species differ in sterile and filtrate-amended gamma-sterilised soils, and are also affected by the presence or absence of the host plant. Thus, it would be desirable to investigate fungus-bacterium interactions using model systems that enable step-wise increases in complexity. The ability to discriminate between different MHB and mycorrhizal fungi will make it possible to obtain a deeper understanding of their interactions when investigating microbial consortia rather than individual species. In the context of the TrophinOak project, we will use the methods presented herein to analyse the responses of AcH 505 and *P. croceum* to soil invertebrates and to investigate how the induction of plant defences affects their abundance.

## Methods

### The soil-based culture system

A soil-based culture system for the quantification of *Streptomyces* sp. AcH 505 and *Piloderma croceum* (DSMZ 4824, ATCC MYA-4870) was established as described by Tarkka et al. [23]. Briefly, micropropagation and rooting of the pedunculate oak clone DF159 (*Quercus robur* L.) were conducted according to Herrmann et al. [47]. Rooted microcuttings were placed in Petri dishes filled with a 1:1 (vol/vol) mixture of fungal inoculum and gamma sterilised soil. Soil filtrates were prepared as described by Rosenberg et al. [48]. At 4 weeks, 5 ml of filtrate was added to the culture system. *Streptomyces* sp. AcH 505, originally isolated from the soil around Norway spruce mycorrhizas in Haigerloch, Germany [18], was maintained on ISP2 agar medium [49]. For AcH 505 treatment, the culture system was inoculated with 2.5 × 10^7^ bacterial spores at 3 and 7 weeks. The material was grown for eight weeks after which bulk soil were harvested from microcosms without plants and bulk as well as rhizosphere samples from microcosms with plants. Rhizosphere samples were taken by harvesting the soil attached to the root. Samples were submerged in liquid nitrogen and stored at −80°C. The experimental design required the analysis of 72 samples in total: 3 (+ oak (rhizosphere/bulk soil)/- oak) × 2 (+/− *P. croceum*) × 2 (+/− AcH 505) × 2 (+/− soil filtrate) × 3 biological replicates.

### DNA extraction

Total DNA was extracted from soil and rhizosphere samples using the PowerSoil DNA Isolation Kit (Mo Bio) according to the manufacturer’s recommendations. The quantity and quality of the DNA were estimated using a Nanodrop spectrophotometer (Thermo Scientific) and agarose gel electrophoresis. For AcH 505 and *P. croceum* pure culture DNA, biological material harvested from liquid culture was immediately frozen in liquid nitrogen (N) and homogenised. DNA extraction was then carried out with the PowerSoil DNA Isolation Kit (Mo Bio) for AcH 505 using a protocol based on those described by P. Spanu (Imperial College, London) and Fulton et al. [50] (detailed protocol acquired from A. Kohler and F. Martin (INRA Nancy) at “http://1000.fungalgenomes.org/home/wp-content/uploads/2012/03/Martin_genomicDNAextraction_AK051010.pdf”) for *P. croceum*.

### Primer design and validation for qRT-PCR

Primers for the quantification of AcH 505 and *P. croceum* were designed using the Primer3 software package [51]http://frodo.wi.mit.edu/primer3/. The designed primer pairs were required to have: a melting temperature of 55–65°C, a GC content of 58 to 63%, primer lengths of 18–22 bp, and amplified product lengths of 70–150 bp. The AcH 505 primers were designed based on genome sequence data (T. Wu., F. B., L. F., M. T. T., unpublished). The ITS region of *P. croceum* (NCBI, JX174048), as well as genomic data for *P. croceum* (Fungal Genomics program, DOE Joint Genome Institute), were used as templates for fungal primer design. The amplicon sizes and sequences for the primers used in this work are listed in Table 1. The identities of the amplified products were verified by Sanger-sequencing.

**Table 1:** **Sequence, expected amplicon sizes, and annealing temperature for the AcH 505 and *P. croceum *primers**

**Target**	**Amplicon size (bp)**	**Primer sequence (5′** → **3′)**	**Annealing temp. (°C)**
AcH 505, intergenic region between *gyrA*/*gyrB* genes	107	AcH107-f (GGCAAGCAGAACGGTAAGCGG)	55
		AcH107-r (TGGTCGGTGTCCATCGTGGT)	
*P. croceum*, ITS	121	ITSP1-f (GGATTTGGAGCGTGCTGGCGT)	55
		ITSP1-r (TTGTGAGCGGGCTTTTCGGACC)	
*P. croceum*, intergenic region between two ORFs	127	Pilo127-f (GTCAGAGACGGACGCAGTTG)	62
		Pilo127-r (CCAGTCAGCGGAGGAGAA)	

The constructed primers were initially used in PCR amplifications to test their functionality and to verify the predicted size of the amplicons. The specificity and the efficiency of the primer pairs was verified by melting curves and the construction of standard curves based on a serial two-fold dilution (2^0^ - 2^-5^) using soil DNA as the template. Template plasmids were used to generate a standard curve that was used as an external standard. The target DNA sequence was cloned into the pGEM-T vector (Promega) and the resulting plasmids were purified. All plasmids were quantified by spectrometry using a Nanodrop ND-1000 instrument (Thermo Scientific) and copy numbers were estimated based on the molecular weight of the template. The number of copies of the cloned target DNA in the dilution series ranged from 10^6^ to 10^1^.

### Real-Time PCR assays

Real-time PCR was performed using the iQ SYBR Green Supermix (Bio-Rad). The reaction mixtures contained 7.5 μl of iQ SYBR Green Supermix, 1 μl of DNA solution (corresponding to 1 ng of DNA), and 350 nmol of each gene-specific primer. The experiments were conducted in 96-well plates with an iQ 5 Multicolour Real-Time PCR Detection System (Bio-Rad). PCR was always performed with three biological and three technical replicates. The cycling conditions were 10 s at 95°C, 30 s at 55°C or 62°C. Template abundances were determined based on the Ct values (which measure the number of cycles at which the fluorescent signal exceeds the background level and surpasses the threshold established based on the exponential phase of the amplification plot). The significance of differences between the Ct values of different treatments were determined by one way analyses of variance ( p < 0.05) and grouped according to the Tukey HSD test in R (R Core team, 2012).

## Competing interests

The authors declare that they have no competing interests.

## Authors’ contributions

FK conducted the molecular studies and drafted the manuscript. KZ participated in the quantification experiments. LF performed the AcH 505 genome assembly. TRN helped with the confocal laser scanning microscopy. TWe did the GFP labelling of AcH 505. VK participated in the electron scanning microscopy studies. TWu carried out the AcH 505 genome sequencing. SH coordinated the establishment of microcosms with oak microcuttings within the TrophinOak platform. FB is the lead scientist of the TrophinOak project. MT conceived of the study, participated in its design and coordination, assisted in the sequencing of the AcH 505 genome and helped to draft the manuscript. All authors read and approved the final manuscript.

## Supplementary Material

Additional file 1 **Experimental setup for quantification of AcH 505 and *P. croceum *under different culture conditions.** Click here for file

Additional file 2 qRT-PCR melting and standard curves obtained using the AcH107 primer pair. Click here for file

Additional file 3 qRT-PCR melting and standard curves obtained with the ITS-P primer pair. Click here for file

Additional file 4 qRT-PCR melting and standard curves obtained with the ITSP1 primer pair. Click here for file

Additional file 5 qRT-PCR melting and standard curves obtained with the Pilo127 primer pair. Click here for file

Additional file 6 **Correlation of AcH 505 and *P. croceum *biomass with qRT-PCR data.** Click here for file

Additional file 7 **Statistical analysis relating to the quantification of the mycorrhization helper bacterium *Streptomyces *sp. AcH 505 and the mycorrhizal fungus *Piloderma croceum *in soil microcosms.** Click here for file

Additional file 8 Cryo-field emission scanning electron microscopy (cryo-FESEM) images. Click here for file

Additional file 9 Confocal laser scanning microscopy (CLSM) images. Click here for file

Additional file 10 **eGFP labelling of *Streptomyces sp. *AcH 505.** Click here for file

Additional file 11 **Visualisation of the *Streptomyces sp. *AcH 505 – *Piloderma croceum *interaction using confocal laser scanning microscopy.** Click here for file
