# Supplementary material for: Detection and quantification of a mycorrhization helper bacterium and a mycorrhizal fungus in plant-soil microcosms at different levels of complexity
Source: BMC Microbiol. 2013 Sep 11;13:205. doi: 10.1186/1471-2180-13-205 (PMC3848169; doi:10.1186/1471-2180-13-205)
Supplement: Additional file 1 — Experimental setup for quantification of AcH 505 and P. croceum under different culture conditions. [file 1471-2180-13-205-S1.pdf]

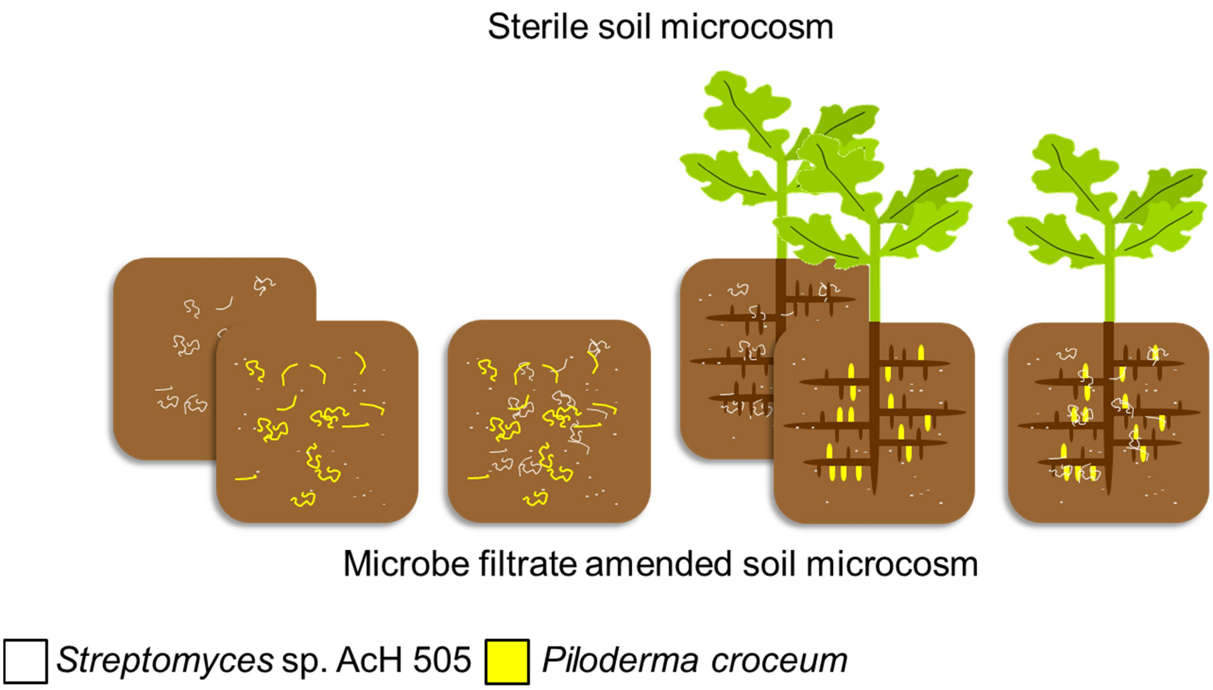

Additional file 1 Experimental setup for quantification of AcH 505 and *P. croceum* under different culture conditions.
