# Supplementary material for: Detection and quantification of a mycorrhization helper bacterium and a mycorrhizal fungus in plant-soil microcosms at different levels of complexity
Source: BMC Microbiol. 2013 Sep 11;13:205. doi: 10.1186/1471-2180-13-205 (PMC3848169; doi:10.1186/1471-2180-13-205)
Supplement: Additional file 2 — qRT-PCR melting and standard curves obtained using the AcH107 primer pair. [file 1471-2180-13-205-S2.pdf]

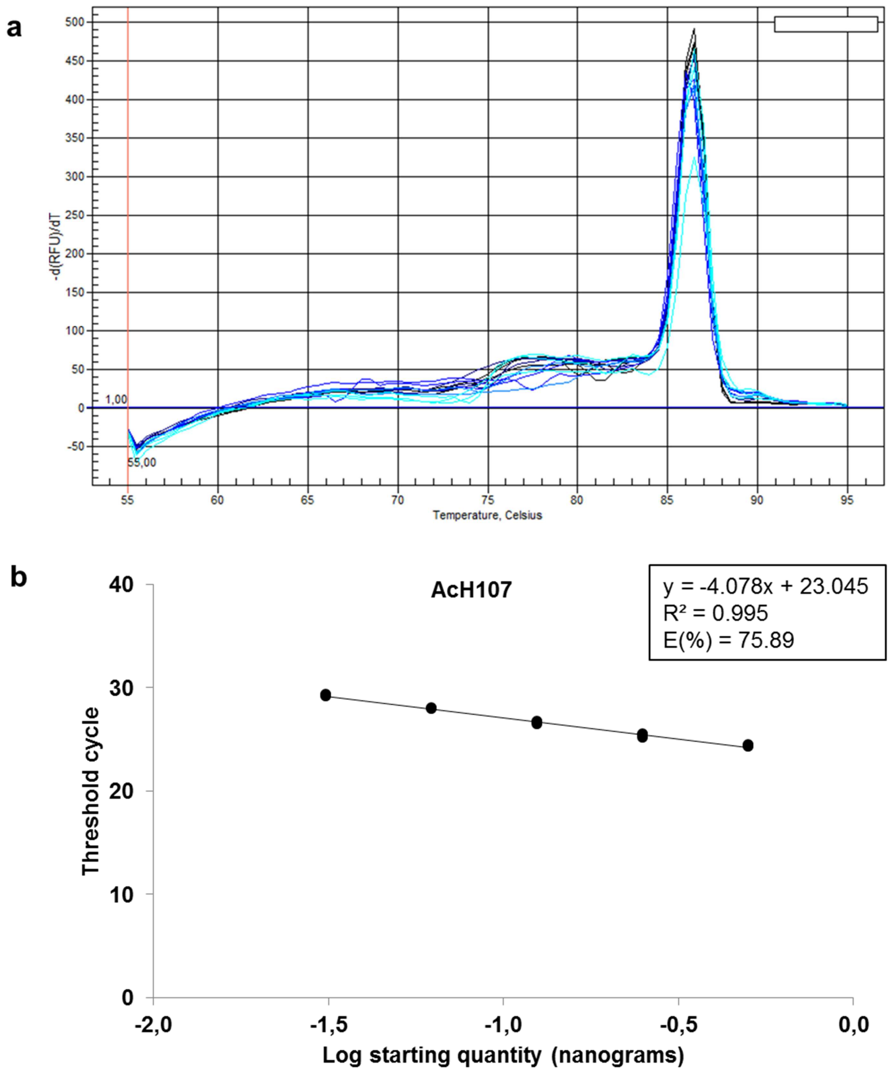

Additional file 2 Standard curves obtained from quantification of *Streptomyces* sp. AcH 505 from non-sterile soil microcosm by the primer pair AcH107. (a) Melting curve of the qPCR amplicon generated from soil DNA. Specific amplification of AcH 505 is indicated by the single sharp peak. (b) Real-time PCR standard curve obtained by amplification of a serial dilution of soil DNA. The curve was generated by plotting the Ct values against the dilutions. The high R^2 value suggests linear amplification.
