# Supplementary material for: Detection and quantification of a mycorrhization helper bacterium and a mycorrhizal fungus in plant-soil microcosms at different levels of complexity
Source: BMC Microbiol. 2013 Sep 11;13:205. doi: 10.1186/1471-2180-13-205 (PMC3848169; doi:10.1186/1471-2180-13-205)
Supplement: Additional file 4 — qRT-PCR melting and standard curves obtained with the ITSP1 primer pair. [file 1471-2180-13-205-S4.pdf]

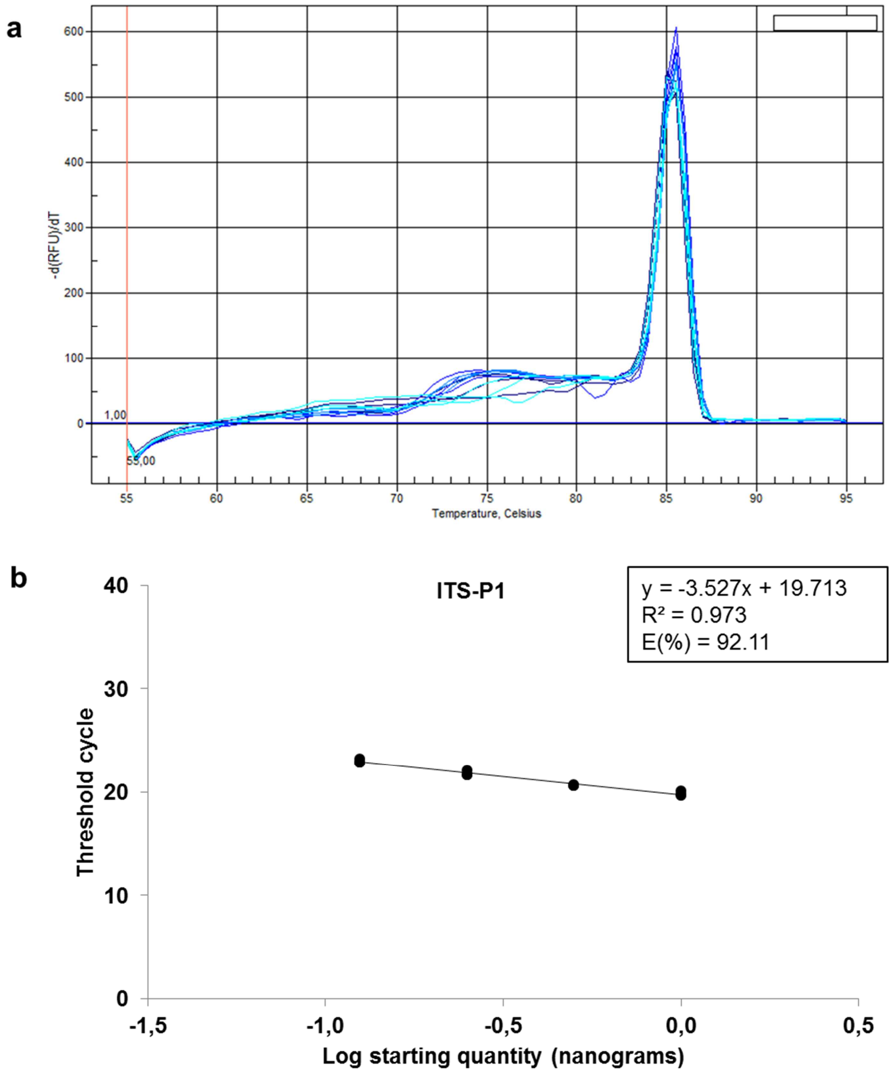

Additional file 4 Standard curves obtained from quantification of *Piloderma croceum* from non-sterile soil microcosm by the primer pair ITSP1. (a) Melting curve of the qPCR amplicon generated from soil DNA. Specific amplification of *P. croceum* is indicated by the single sharp peak. (b) Real-time PCR standard curve obtained by amplification of a serial dilution of soil DNA. The curve was generated by plotting the Ct values against the dilutions. The high R^2 value suggests linear amplification.
