# Supplementary material for: Detection and quantification of a mycorrhization helper bacterium and a mycorrhizal fungus in plant-soil microcosms at different levels of complexity
Source: BMC Microbiol. 2013 Sep 11;13:205. doi: 10.1186/1471-2180-13-205 (PMC3848169; doi:10.1186/1471-2180-13-205)
Supplement: Additional file 6 — Correlation of AcH 505 and P. croceum biomass with qRT-PCR data. [file 1471-2180-13-205-S6.pdf]

Additional file 6 Correlation of AcH 505 and *P. croceum* biomass with qRT-PCR data. 150 and 250 mg mycelium were extracted in a final volume of 100 and 1000 µl for AcH 505 and *P. croceum*, respectively. For qRT-PCR reactions with 1 µl of undiluted to a dilution of 10^{-5} yielded good amplification of the product. The initial copy number was derived from the plasmid standard curve (Figure 2).

	Mycelium (mg)	Initial copy number	Mycelium (mg) / copy number
AcH505 - 107f/r	1,50E+00	8,97E+05	1,67E-06
	1,50E-01	1,17E+05	1,28E-06
	1,50E-02	1,18E+04	1,27E-06
	1,50E-03	1,35E+03	1,11E-06
	1,50E-04	2,69E+02	5,57E-07
	1,50E-05	1,32E+01	1,14E-06
P.croceum – ITS-P1	2,50E-01	7,58E+06	3,30E-08
	2,50E-02	7,74E+05	3,23E-08
	2,50E-03	7,89E+04	3,17E-08
	2,50E-04	5,24E+03	4,77E-08
	2,50E-05	5,77E+02	4,34E-08
	2,50E-06	4,54E+01	5,51E-08
P.croceum – 127f/r	2,50E-01	8,33E+05	3,00E-07
	2,50E-02	8,85E+04	2,83E-07
	2,50E-03	9,35E+03	2,67E-07
	2,50E-04	5,50E+02	4,54E-07
	2,50E-05	7,45E+01	3,36E-07
	2,50E-06	9,99E+00	2,50E-07
