# Supplementary material for: Detection and quantification of a mycorrhization helper bacterium and a mycorrhizal fungus in plant-soil microcosms at different levels of complexity
Source: BMC Microbiol. 2013 Sep 11;13:205. doi: 10.1186/1471-2180-13-205 (PMC3848169; doi:10.1186/1471-2180-13-205)
Supplement: Additional file 7 — Statistical analysis relating to the quantification of the mycorrhization helper bacterium Streptomyces sp. AcH 505 and the mycorrhizal fungus Piloderma croceum in soil microcosms. [file 1471-2180-13-205-S7.pdf]

Additional file 7 Statistical analysis of quantification of mycorrhization helper bacterium *Streptomyces* sp. AcH 505 and the mycorrhizal fungus *Piloderma croceum* in soil microcosms. p-values determined by Tukey HSD test for statistical analyses of quantification results (n.s. = not significant)

Primer	Treatment	p-value
AcH107 without plant	AcH505 - AcH505 + Filtrat	n.s.
	AcH505 - AcH505 + *P.croceum*	$p < 0.001$
	AcH505 - AcH505 + Filtrat + *P.croceum*	$p < 0.001$
	AcH505 + Filtrat - AcH505 + *P.croceum*	$p < 0.001$
	AcH505 + Filtrat - AcH505 + Filtrat + *P.croceum*	$p < 0.001$
	AcH505 + *P.croceum* - AcH505 + Filtrat + *P.croceum*	$p < 0.05$
AcH107 with plant rhizosphere	AcH505 - AcH505 + Filtrat	n.s.
	AcH505 - AcH505 + *P.croceum*	n.s.
	AcH505 - AcH505 + Filtrat + *P.croceum*	$p < 0.001$
	AcH505 + Filtrat - AcH505 + *P.croceum*	$p < 0.01$
	AcH505 + Filtrat - AcH505 + Filtrat + *P.croceum*	$p < 0.001$
	AcH505 + *P.croceum* - AcH505 + Filtrat + *P.croceum*	$p < 0.01$
AcH107 with plant - bulk soil	AcH505 - AcH505 + Filtrat	$p < 0.01$
	AcH505 - AcH505 + *P.croceum*	$p < 0.001$
	AcH505 - AcH505 + Filtrat + *P.croceum*	$p < 0.001$
	AcH505 + Filtrat - AcH505 + *P.croceum*	$p < 0.001$
	AcH505 + Filtrat - AcH505 + Filtrat + *P.croceum*	$p < 0.001$
	AcH505 + *P.croceum* - AcH505 + Filtrat + *P.croceum*	n.s.
PiloITS without plant	*P.croceum* - *P.croceum* + Filtrat	$p < 0.05$
	P.croceum - *P.croceum* + AcH505	n.s.
	P.croceum - *P.croceum* + Filtrat + AcH505	n.s.
	P.croceum + Filtrat - *P.croceum* + AcH505	$p < 0.01$
	P.croceum + Filtrat - *P.croceum* + Filtrat + AcH505	n.s.
	P.croceum + AcH505 - *P.croceum* + Filtrat + AcH505	n.s.
PiloITS with plant - rhizosphere	*P.croceum* - *P.croceum* + Filtrat	$p < 0.001$
	P.croceum - *P.croceum* + AcH505	n.s.
	P.croceum - *P.croceum* + Filtrat + AcH505	$p < 0.001$
	P.croceum + Filtrat - *P.croceum* + AcH505	$p < 0.001$
	P.croceum + Filtrat - *P.croceum* + Filtrat + AcH505	$p < 0.001$
	P.croceum + AcH505 - *P.croceum* + Filtrat + AcH505	$p < 0.001$
PiloITS with plant - bulk soil	*P.croceum* - *P.croceum* + Filtrat	$p < 0.01$
	P.croceum - *P.croceum* + AcH505	n.s.
	P.croceum - *P.croceum* + Filtrat + AcH505	$p < 0.001$
	P.croceum + Filtrat - *P.croceum* + AcH505	$p < 0.05$
	P.croceum + Filtrat - *P.croceum* + Filtrat + AcH505	n.s.
	P.croceum + AcH505 - *P.croceum* + Filtrat + AcH505	$p < 0.01$
Pilo127 without plant	*P.croceum* - *P.croceum* + Filtrat	$p < 0.05$

	P.croceum - *P.croceum* + AcH505	n.s.
	P.croceum - *P.croceum* + Filtrat + AcH505	n.s.
	P.croceum + Filtrat - *P.croceum* + AcH505	n.s.
	P.croceum + Filtrat - *P.croceum* + Filtrat + AcH505	n.s.
	P.croceum + AcH505 - *P.croceum* + Filtrat + AcH505	n.s.
Pilo127 with plant - rhizosphere	*P.croceum* - *P.croceum* + Filtrat	$p < 0.001$
	P.croceum - *P.croceum* + AcH505	n.s.
	P.croceum - *P.croceum* + Filtrat + AcH505	$p < 0.001$
	P.croceum + Filtrat - *P.croceum* + AcH505	$p < 0.001$
	P.croceum + Filtrat - *P.croceum* + Filtrat + AcH505	$p < 0.01$
	P.croceum + AcH505 - *P.croceum* + Filtrat + AcH505	$p < 0.001$
Pilo127 with plant - bulk soil	*P.croceum* - *P.croceum* + Filtrat	$p < 0.05$
	P.croceum - *P.croceum* + AcH505	n.s.
	P.croceum - *P.croceum* + Filtrat + AcH505	n.s.
	P.croceum + Filtrat - *P.croceum* + AcH505	n.s.
	P.croceum + Filtrat - *P.croceum* + Filtrat + AcH505	n.s.
	P.croceum + AcH505 - *P.croceum* + Filtrat + AcH505	n.s.
