# Supplementary material for: Detection and quantification of a mycorrhization helper bacterium and a mycorrhizal fungus in plant-soil microcosms at different levels of complexity
Source: BMC Microbiol. 2013 Sep 11;13:205. doi: 10.1186/1471-2180-13-205 (PMC3848169; doi:10.1186/1471-2180-13-205)
Supplement: Additional file 8 — Cryo-field emission scanning electron microscopy (cryo-FESEM) images. [file 1471-2180-13-205-S8.pdf]

Additional file 8 Cryo-field emission scanning electron microscopy (cryo-FESEM)

Samples were transferred into a special cryo holder, mounted into a drop of glue and clamped. The samples were quickly frozen (>10^3 K/s) in slushy nitrogen. Then, the samples were transferred into the cryo-stage of the preparation chamber (ALTO2500, Gatan, USA) where they were freeze-fractured at -140 °C, freeze-etched at -95 °C for 3 min, and then coated with 3 nm layer of platinum at -135 °C. The coated samples were inserted into the chamber of the JSM-7401F microscope (JEOL, Japan) precooled to -130 °C. Images were obtained by both the secondary and back-scattered electron signal at 3 kV.
