# Supplementary material for: Detection and quantification of a mycorrhization helper bacterium and a mycorrhizal fungus in plant-soil microcosms at different levels of complexity
Source: BMC Microbiol. 2013 Sep 11;13:205. doi: 10.1186/1471-2180-13-205 (PMC3848169; doi:10.1186/1471-2180-13-205)
Supplement: Additional file 9 — Confocal laser scanning microscopy (CLSM) images. [file 1471-2180-13-205-S9.pdf]

Additional file 9 Confocal laser scanning microscopy (CLSM)

For CLSM imaging of GFP labelled bacteria on roots a SP1 (Leica, Wetzlar) controlled by the confocal software version 2.61 built 1537 was used. The upright microscope was equipped with three lasers (Ar, DPSS561, HeNe). Samples were mounted in a coverwell chamber and examined with a 63x NA 1.2 wi objective lens. For excitation the Ar laser line at 488 nm was used, emission signals were detected from 500-600 nm. Optical sections were recorded at 0.2 µm stepsize with a frame average of eight. Image series are presented as maximum intensity projection using the microscope software.
