# Supplementary material for: Detection and quantification of a mycorrhization helper bacterium and a mycorrhizal fungus in plant-soil microcosms at different levels of complexity
Source: BMC Microbiol. 2013 Sep 11;13:205. doi: 10.1186/1471-2180-13-205 (PMC3848169; doi:10.1186/1471-2180-13-205)
Supplement: Additional file 10 — eGFP labelling of Streptomyces sp. AcH 505. [file 1471-2180-13-205-S10.pdf]

Additional file 10 eGFP labelling of *Streptomyces sp.* AcH 505

To obtain eGFP labelled *S. sp.* AcH505, the plasmid pRM4.3, which is a pSET152 derivative containing the *egfp* gene under control of the constitutive ermE* promoter [1] was introduced into the strain by interspecific conjugation using a modified protocol according to Kieser et al. [2]: E. coli ET12567 (pUB307) [3, 4] was transformed with pRM4.3. Transformants were grown over night in LB supplemented with the antibiotics kanamycin 50 µg x mL $^{-1}$, chloramphenicol 12.5 µg x mL^{-1}, and apramycin 50 µg x mL^{-1} at 37 °C on a rotary shaker. Cells were harvested, washed twice with LB without antibiotics and resuspended in 1 ml of LB. 100 µL of this cell suspension was mixed with ~ 1x 10^{8} spores of *S. sp.* AcH 505 and plated on SFM medium 20 % soybean flour, 20 % mannitol, 1.6 % agar, pH 7.5) supplemented with 10 mM $MgCl_2$. After 16 h cultivation at 29 °C, the plates were overlayed with 1 ml H_2O containing 1.5 mg x mL^{-1} apramycin and 0.75 mg x mL^{-1} nalidixic acid.

References

1. Chevillotte M, Menges R, Muth G, Wohlleben W, Stegmann E: **A quick and reliable method for monitoring gene expression in actinomycetes**. *J Biotechnol* 2008, **135**:262-265.

2. Kieser T, Bibb MJ, Buttner MJ, Chater KF, Hopwood DA: **Practical *Streptomyces* Genetics**. 2000.

3. MacNeil DJ, Gewain KM, Ruby CL, Dezeny G, Gibbons PH, MacNeil T: **Analysis of *Streptomyces avermitilis* genes required for avermectin biosynthesis utilizing a novel integration vector**. *Gene* 1992, **111**:61-68.

4. Flett F, Mersinias V, Smith CP: **High efficiency intergeneric conjugal transfer of plasmid DNA from *Escherichia coli* to methyl DNA-restricting streptomycetes**. *FEMS Microbiol Lett* 1997, **155**:223-229.
