# Supplementary material for: Detection and quantification of a mycorrhization helper bacterium and a mycorrhizal fungus in plant-soil microcosms at different levels of complexity
Source: BMC Microbiol. 2013 Sep 11;13:205. doi: 10.1186/1471-2180-13-205 (PMC3848169; doi:10.1186/1471-2180-13-205)
Supplement: Additional file 11 — Visualisation of the Streptomyces sp. AcH 505 – Piloderma croceum interaction using confocal laser scanning microscopy. [file 1471-2180-13-205-S11.pdf]

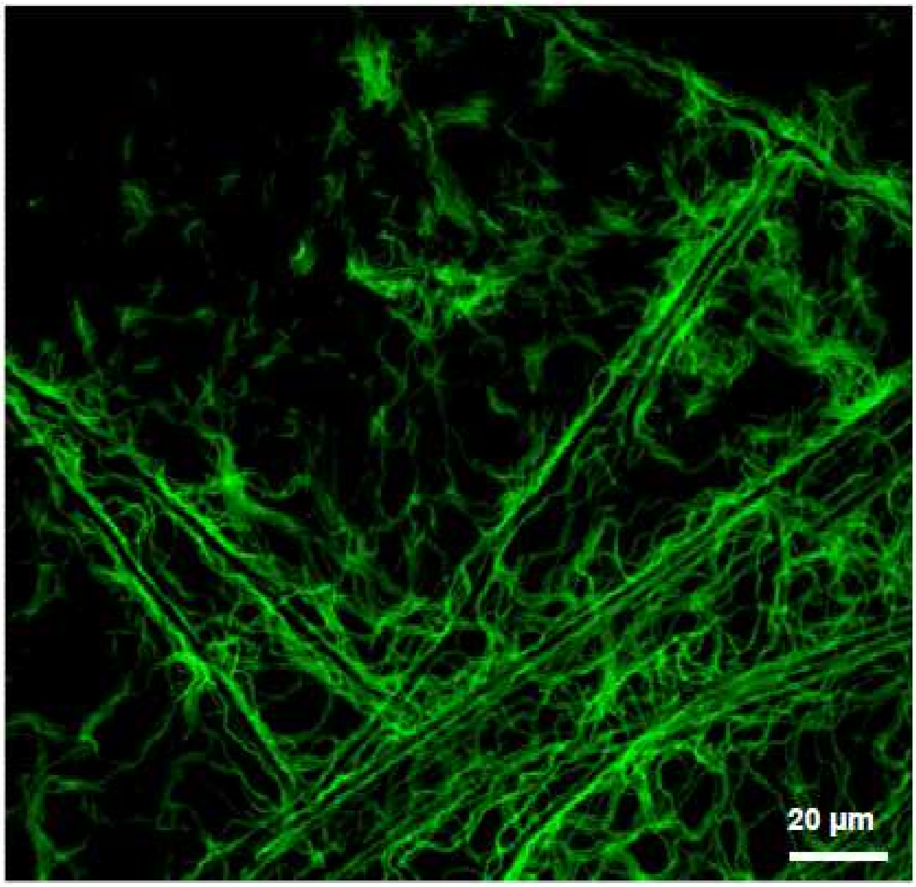

Additional file 11 Visualisation of the *Streptomyces sp*. AcH 505 – *Piloderma croceum* interaction using confocal laser scanning microscopy. *Streptomyces sp*. AcH 505 was labelled with Green Fluorescent Protein.
